# College and Hospital Notes

**Published:** 1900-05

**Authors:** 


### COLLEGE AND HOSPITAL NOTES.

THE Jefferson College Hospital, at Philadelphia, together with the handful of dilapidated buildings that complete the rectangular lot of ground upon which the hospital now stands will be torn down soon. On that site in the heart of the city, will be erected a spacious modern hospital building, constructed after the best features of the leading hospitals of the world. In connection with it will be established a maternity hospital, larger than anything of its kind in Philadelphia; also a training school for women nurses and a training school for men nurses.

The committee which has had the new hospital in hand is composed of six gentlemen, and Mr. Edward H Weil, president of the Board of Trustees, is chairman. Last February they issued a circular to all the departments of both the college and hospital, asking information that would assist in the preparation of plans. A visiting committee has gone over all the hospitals in this part of the country to investigate the latest improvements and report.

It will be gratifying to the many alumni and friends of Jefferson throughout the world to learn of the prospects for a new structure of imposing proportions, that is on the eve of materialisation.

---

THE Margaret Fahnestock Training School for Nurses, No. 304 East Twentieth Street, New York was opened with impressive ceremonies, April 19, 1900.

The building was erected by Harris C. Fahnestock as a memorial to his wife who died in 1898. In her long illness she was attended by nurses from the Post-Graduate Hospital, whose devotion, with that of the physicians, was the cause of Mr. Fahnestock's resolve to put his memorial to his wife in a form that should be a helpful agency to all nurses.

He accordingly bought a large property in East Twentieth Street, opposite the hospital, and on May 1, 1899, the building was begun. It is a large six-story building, in the old Dutch Colonial style of architecture, and is designed to serve as a home for the nurses in the training school connected with the hospital.

All the appointments of the building are artistic as well as scientific. The entrance floor contains a large reception hall finished in white, with Pompeiian red walls. Over the fireplace is a large bronze tablet bearing this memorial inscription:

IN MEMORY OF
MARGARET A. FAHNESTOCK,
September 3, 1835.
December 2, 1898.

Two large reception rooms in sage green and mahogany and a lecture room that seats 175 persons occupy the remainder of the floor. Above this are the apartments for the nurses, every one having a separate room. Provision is made for between sixty and seventy, and in addition are the "invalid rooms," with their separate bathrooms and cooking room. These can be completely isolated from the rest of the house by the arrangement of doors and corridors.

Every floor of sleeping rooms has four bathrooms, and the finish throughout is in accord with the latest sanitary methods. The rooms are all prettily furnished with white enamel in colonial style, and handsome rugs cover the floors. An important feature is the roof garden, with its well-stocked wall boxes of thriving plants. Here are many rocking chairs and hammocks, and couches will be added as the weather makes it the evening resort.

The exercises at the opening were simple and brief. Addresses were made by the Rev. Dr. William B. Bodine, of Philadelphia, and Warner Van Norden, after which Mr. Fahnestock made the presentation of the necessary legal deeds. Dr. D. B. St. John Roosa accepting them as president of the hospital corporation. The many guests then inspected the house.

THE New York State Hospital for the treatment of incipient tuberculosis is soon to be established in the Adirondacks. A bill appropriating $50,000 with which to begin the work passed the last legislature and Governor Roosevelt recently appointed five trustees, who under the act, will locate the hospital and perform such other duties as will put the plan in operation. The names of the trustees are as follows: To serve five years Howard Townsend, of New York; four years, Dr. John H. Pryor, of Buffalo; three years, Dr. Willis G. Macdonald, of Albany; two years, Walter Jennings, of New York; one year, Frank E. Kendall, of Saranac Lake. The trustees are to purchase 1,000 acres of land for the site and the state forest and preserve board is to set apart a like amount of state land—the location to be approved jointly by the state board of health and the state forest and preserve board. It is expected that the board will meet at Albany early in May for organisation and conference.

